# Computational prediction of protein subdomain stability in *MYBPC3* enables clinical risk stratification in hypertrophic cardiomyopathy and enhances variant interpretation

**DOI:** 10.1038/s41436-021-01134-9

**Published:** 2021-03-29

**Authors:** Andrea D. Thompson, Adam S. Helms, Anamika Kannan, Jaime Yob, Neal K. Lakdawala, Samuel G. Wittekind, Alexandre C. Pereira, Daniel L. Jacoby, Steven D. Colan, Euan A. Ashley, Sara Saberi, James S. Ware, Jodie Ingles, Christopher Semsarian, Michelle Michels, Francesco Mazzarotto, Iacopo Olivotto, Carolyn Y. Ho, Sharlene M. Day

**Affiliations:** 1 Cardiovascular Medicine, University of Michigan, Ann Arbor, MI USA; 2 Cardiovascular Medicine, University of Pennsylvania, Philadelphia, PA USA; 3 Cardiovascular Medicine, Brigham and Women’s Hospital, Harvard Medical School, Boston, MA USA; 4 Cincinnati Children’s Hospital Medical Center, Heart Institute, Cincinnati, OH USA; 5 Heart Institute (InCor), University of Sao Paolo Medical School, Sao Paulo, Brazil; 6 Cardiovascular Medicine, Yale University, New Haven, CT USA; 7 Department of Cardiology, Boston Children’s Hospital, Boston, MA USA; 8 Center for Inherited Heart Disease, Stanford University, Stanford, CA USA; 9 National Heart & Lung Institute & Royal Brompton Cardiovascular Research Centre, Imperial College London, London, United Kingdom; 10 Cardio Genomics Program at Centenary Institute, The University of Sydney, Sydney, Australia; 11 Agnes Ginges Centre for Molecular Cardiology at Centenary Institute, The University of Sydney, Sydney, Australia; 12 Department of Cardiology, Thoraxcenter, Erasmus MC Rotterdam, The Netherlands; 13 Cardiomyopathy Unit, Careggi University Hospital, Florence, Italy; 14 Department of Clinical and Experimental Medicine, University of Florence, Florence, Italy

## Abstract

**Purpose:**

Variants in *MYBPC3* causing loss of function are the most common cause of hypertrophic cardiomyopathy (HCM). However, a substantial number of patients carry missense variants of uncertain significance (VUS) in *MYBPC3*. We hypothesize that a structural-based algorithm, STRUM, which estimates the effect of missense variants on protein folding, will identify a subgroup of HCM patients with a *MYBPC3* VUS associated with increased clinical risk.

**Methods:**

Among 7,963 patients in the multicenter Sarcomeric Human Cardiomyopathy Registry (SHaRe), 120 unique missense VUS in *MYBPC3* were identified. Variants were evaluated for their effect on subdomain folding and a stratified time-to-event analysis for an overall composite endpoint (first occurrence of ventricular arrhythmia, heart failure, all-cause mortality, atrial fibrillation, and stroke) was performed for patients with HCM and a *MYBPC3* missense VUS.

**Results:**

We demonstrated that patients carrying a *MYBPC3* VUS predicted to cause subdomain misfolding (STRUM+, ΔΔG ≤ −1.2 kcal/mol) exhibited a higher rate of adverse events compared with those with a STRUM- VUS (hazard ratio = 2.29, *P* = 0.0282). In silico saturation mutagenesis of *MYBPC3* identified 4,943/23,427 (21%) missense variants that were predicted to cause subdomain misfolding.

**Conclusion:**

STRUM identifies patients with HCM and a *MYBPC3* VUS who may be at higher clinical risk and provides supportive evidence for pathogenicity.

## INTRODUCTION

Genetic variant interpretation is an ongoing challenge in clinical medicine, particularly when the gene of interest lacks robust functional assays.^1,2^ A variety of computational algorithms have been developed to predict variant pathogenicity, but their sensitivity and specificity are often poor, particularly when applied broadly across different diseases and different genes.^1,3^ Loss-of-function (LoF) pathogenic variants are common,^1,4,5^ resulting from either frameshift or nonsense variants creating a premature stop codon, splice errors, disruption of enzymatic activity, alteration of protein–protein interactions, or protein misfolding.^1,6,7^ Recognizing a common mechanism by which variants in a particular gene lead to LoF can inform the development of gene-specific computational algorithms to more accurately predict pathogenicity among variants that cannot be confidently classified based on clinical and family data alone.^6,7^

Herein we focus on *MYBPC3* (encoding the protein, cardiac myosin binding protein C, or MyBP-C). Pathogenic variants in *MYBPC3* account for ~50% of patients with sarcomeric hypertrophic cardiomyopathy (HCM),^8,9^ and are inherited in an autosomal dominant fashion (OMIM 115197). Patients with HCM can experience a variety of adverse clinical outcomes, including outflow tract obstruction, arrhythmias, heart failure, and sudden cardiac death.^8^ Genetic variants in *MYBPC3* consist of both truncating and nontruncating types. Rarely found in healthy populations, truncating *MYBPC3* variants result in a premature stop codon and cause HCM through complete LoF and haploinsufficiency at the transcript and protein level.^10–13^ Thus, interpretation of these truncating variants as pathogenic is straightforward.^14^

However, the interpretation of missense variants within *MYBPC3* presents a major challenge. Single amino acid substitutions (missense variants) are found commonly in healthy populations. Further, since missense variants do not disrupt the reading frame, protein function may be tolerant to these minor sequence changes. Thus, many missense variants lack sufficient evidence to be classified as either pathogenic or benign and are classified as variants of uncertain significance (VUS).^14,15^ While identifying pathogenic variants allows for predictive genetic testing in at-risk relatives,^16^ a VUS is not clinically actionable and may lead to misinterpretation by clinicians and patients.^17^

Identification of a pathogenic sarcomere genetic variant for HCM also has important prognostic implications. Patients with HCM and a pathogenic sarcomere variant (sarcomeric HCM) have a higher risk of adverse clinical outcomes compared with those without a sarcomere gene variant (nonsarcomeric HCM).^8,18^ Patients carrying a sarcomere gene VUS, on average, exhibit an intermediate risk of adverse events,^8^ most likely because VUS represent a mixed pool of pathogenic and benign variants that cannot be parsed on the basis of clinical and genetic data alone.

Because LoF is an established mechanism for pathogenic variants in *MYBPC3*, we hypothesized that applying a computational approach, called STRUM,^19–21^ that incorporates both sequence-based and structure-based algorithms to missense *MYBPC3* VUS will identify those variants that result in protein subdomain misfolding (STRUM+), thereby supporting pathogenicity and improving variant interpretation. We further predict that this approach will identify a subpopulation of patients with HCM and a STRUM+ *MYBPC3* missense VUS who are at risk for adverse clinical outcomes, at a frequency similar to patients with HCM carrying known pathogenic variants.

## MATERIALS AND METHODS

### Sarcomeric Human Cardiomyopathy Registry (SHaRe) data extraction and *MYBPC3* variant classification

The generation of the centralized SHaRe database has been previously described.^8^ Data were exported from quarter 1 of 2019. Inclusion criteria included a site-designated diagnosis of HCM using standard diagnostic criteria.^8^ SHaRe nontruncating *MYBPC3* missense variants (Tables S1, S2) were classified as previously reported^14^ in accordance with American College of Medical Genetics and Genomics (ACMG) and Association for Molecular Pathology (AMP) joint guidelines, leveraging available clinical and experimental data.^3,8,9,14,22,23^ Known splice variants are classified as truncating. Since variants in *MYBPC3* present in gnomAD with allele frequencies of >4E-05 and absent in SHaRe are unlikely to be independently pathogenic for HCM, these variants were included in our list of benign *MYBPC3* variants.^14^ More details regarding variant interpretation are provided within the Supplemental materials.

It has previously been shown that patients carrying pathogenic nontruncating variants exhibit similar clinical outcomes to those carrying truncating *MYBPC3* variants.^14^ Thus, a reference population including previously adjudicated truncating and nontruncating *MYBPC3* pathogenic/likely pathogenic (pathogenic) variants (*MYBPC3*-path-all) was used. A second reference population included patients with HCM who underwent genetic testing and were negative for sarcomere variants Sarc-.^8^

### Computational structural and protein folding stability predictive modeling

MyBP-C is made up of immunoglobulin and fibronectin subdomains (C0-C10) (NM_000256.3, NP_000247.2). For *MYBPC3* missense variants we utilized STRUM to calculate the effect of the missense variant on the Gibbs free energy of local subdomain folding (ΔΔG)^19^ (Table S3). A negative ΔΔG value indicates the degree of reduced folding energy (kcal/mol) relative to the wild-type subdomain, or folding destabilization.^19^ Previous experimental validation of this algorithm compared STRUM predictions to 3,421 experimentally tested variants from 150 proteins and demonstrated a Pearson’s correlation coefficient of 0.79 and root mean square error of prediction of 1.2 kcal/mol.^19^ Thus, a value of ΔΔG ≤ −1.2 kcal/mol was defined as the cutoff for destabilizing (deleterious) variants. Further details regarding STRUM analysis and structural models are provided within the Supplemental Materials (Figure S1–S3, Table S3).

### Computational sequence-based variant analysis (PolyPhen-2, SIFT, CardioBoost)

We compared the STRUM prediction for *MYBPC3* missense variants with a sequence-based algorithm embedded in STRUM (SIFT).^24,25^ We also analyzed these variants with PolyPhen-2 (HumVar database), another sequence based algorithm.^26^ Finally, we compared our result with those obtained using CardioBoost, which is a disease specific machine learning classifier to predict pathogenicity of rare missense variants in genes associated with cardiomyopathies and arrhythmias.^6^ CardioBoost relies on minor allele frequency, whereas STRUM does not.

### Clinical outcomes analysis

Only patients with HCM carrying a single *MYBPC3* missense VUS were included in clinical outcomes analyses to avoid confounding from cases with multiple gene variants.^27^ Comparisons using time-to-event analysis were made between variants predicted to be deleterious (STRUM+, ΔΔG ≤ −1.2 kcal/mol) and those predicted to be nondeleterious. The primary outcome was an overall composite previously defined as the first occurrence of any component of the ventricular arrhythmia composite, heart failure composite (without inclusions of LV ejection fraction), all-cause mortality, atrial fibrillation (AF), or stroke.^8^ Results were compared with reference populations *MYBPC3*-path-all and Sarc-. A secondary analysis of a heart failure composite, ventricular arrhythmia composite, and atrial fibrillation was also performed. Finally, a secondary analysis using alternative computational algorithms (SIFT, PolyPhen-2, CardioBoost) was performed. Composite outcomes are defined in more detail in the Supplemental materials.

### Statistical analysis

Data presented as mean ± standard deviation were analyzed by *t*-test for two groups or analysis of variance (ANOVA) for >2 groups with Tukey’s post hoc test for multiple comparisons. Data presented as frequency were analyzed by a chi-square test. Odds ratio (with 95% confidence interval [CI]), specificity, and sensitivity were calculated to evaluate the association between computational prediction algorithms and known pathogenic/likely pathogenic (pathogenic) or benign/likely benign (benign) variants (further details provided in supplemental materials). Primary and secondary clinical outcomes were analyzed by the Kaplan–Meier method from time of birth. Analysis from time of birth is appropriate given that the genetic variant is present from birth and variability in time to, and reason for, clinical presentation could confound the results if time from diagnosis were used. Patients who did not have the outcome of interest were censored at the time of their last recorded follow-up in SHaRe. Comparison between curves was performed using Log-rank Mantel–Cox test with *p* values of <0.05 considered statistically significant. Median event free survival and hazard ratio (Mantel–Haenszel) are also reported. Statistical analyses were performed using GraphPad Prism software (San Diego, CA).

## RESULTS

### Patients with HCM and a *MYBPC3* missense VUS predicted to disrupt subdomain folding (STRUM+) exhibit a higher incidence of adverse clinical outcomes

**Fig. 1:**
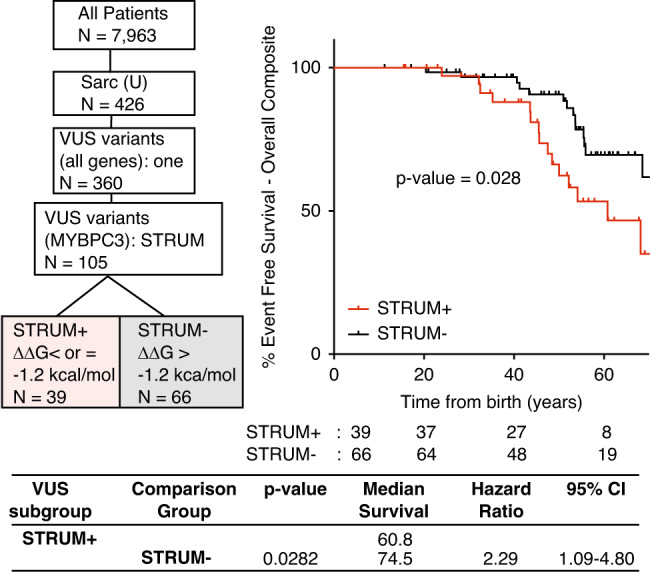
Patients with a *MYBPC3* VUS identified as deleterious by STRUM (STRUM+) are associated with an increased risk for adverse hypertrophic cardiomyopathy (HCM)-related outcomes. Selection within Sarcomeric Human Cardiomyopathy Registry (SHaRe) of patients with HCM carrying a single *MYBPC3* missense variant of uncertain significance (VUS) is shown on the left. One hundred five patients carry a single MYBPC3 *missense* VUS, covering 77 distinct *MYBPC3* VUS. Kaplan–Meier curves, median event free survival (years), and hazard ratio with corresponding 95% confidence interval (CI) reveal that patients carrying a STRUM+ *MYBPC3* VUS (red) exhibited a higher rate of adverse HCM-related outcomes (overall composite) compared with patients carrying a STRUM- variant (black).

**Fig. 2:**
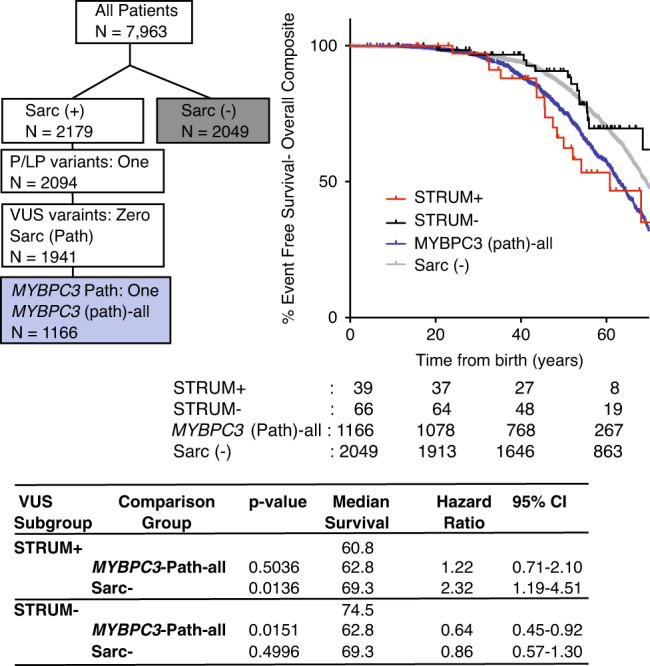
Patients with a *MYBPC3* variant of uncertain significance (VUS) identified as deleterious by STRUM (STRUM+) exhibit clinical outcomes similar to patients with a *MYBPC3* pathogenic variants. Selection within Sarcomeric Human Cardiomyopathy Registry (SHaRe) of patients with hypertrophic cardiomyopathy (HCM) and a single *MYBPC3* pathogenic variant (*MYBPC3* -Path-all) and patients with HCM without a sarcomere gene variant after clinical genotype analysis (Sarc−) is shown on the left. Kaplan–Meier curves, median event free survival (median survival), and hazard ratio with corresponding 95% confidence interval (CI) reveal patients carrying a STRUM+ *MYBPC3* VUS (red) exhibited overall composite outcomes similar to *MYBPC3*-Path-all patients (blue, *p* value 0.5036). Whereas, patients carrying a STRUM- variant (black) exhibited a lower rate of adverse HCM-related outcomes (overall composite) similar to Sarc- patients (gray).

We began by evaluating all *MYBPC3* missense VUS within SHaRe using STRUM. *MYBPC3* VUS exhibited a mean ΔΔG of −0.73 ± 1.06 kcal/mol (Figure S4). Of 120 unique *MYBPC3* missense VUS, 34 (28%) were predicted to cause subdomain misfolding with ΔΔG values ≤ −1.2 kcal/mol (deleterious) (Table S2). Next, we evaluated clinical characteristics and outcomes in patients with HCM and a single missense *MYBPC3* VUS predicted to disrupt subdomain folding (STRUM+) compared with patients carrying a VUS not predicted to disrupt subdomain folding (STRUM-). For this analysis, we included only patients who carried a single VUS within *MYBPC3*, and excluded patients who carried a second pathogenic variant or variant of uncertain significance (*N* = 105). Patients with a STRUM+ versus STRUM- *MYBPC3* VUS exhibited similar clinical characteristics including body mass index (BMI), gender, ancestry, age at diagnosis, wall thickness, ejection fraction, and left ventricular outflow tract obstruction (Table 1). We observed that patients carrying a STRUM+ VUS experienced higher rates of adverse events compared with patients carrying a STRUM- VUS (Fig. 1, hazard ratio 2.3, *p* = 0.03). Furthermore, patients carrying a STRUM+ VUS exhibited a similar rate of adverse clinical events compared with patients carrying a pathogenic variant (*MYBPC3*-Path-all). Conversely, patients carrying STRUM- VUS exhibited a lower frequency of outcomes, similar to Sarc- patients (Fig. 2). There were no statistically significant differences between groups for the individual component outcomes, including ventricular arrhythmias, heart failure, or atrial fibrillation (Figure S5).

**Table 1:** Demographics of patients with HCM and single *MYBPC3* VUS.

	STRUM (+) *n* = 39 (37%)	STRUM (−) *n* = 66 (63%)	*p* value STRUM (+) vs. STRUM (−)
**Baseline characteristics**			
Female, *n* (%)	10 (26%)	14 (21%)	0.6308
Age at diagnosis, mean (STD), year	40.31 (17.13)	41.34 (21.17)	0.9930
Follow-up time, mean (STD), year	9.82 (10.00)	12.18 (11.83)	0.7575
Maximum BMI, mean (STD) kg/m^2^	28.87 (4.10)	27.96 (6.03)	0.9390
lb/ft^2^	685.09 (97.29)	663.49 (143.09)	
Race, *n* %			0.5107
White	35 (90%)	60 (91%)	
Black	2 (5%)	1 (2%)	
Other/not reported	2 (5%)	5 (8%)	
Proband, *n* (%)	35 (90%)	64 (97%)	0.1232
Family history HCM, *n* (%)	13 (33%)	19 (29%)	0.6249
Family history SCD *n* (%)	8 (21%)	9 (14%)	0.3553
**Echocardiogram data**			
Maximal LVWT, mean (STD), mm	22.73 (6.97)	20.83 (7.29)	0.4287
Minimum LVEF, mean (STD), %	59.88 (8.32)	60.12 (11.34)	0.9996
LVOT peak gradient ≥30 mmHg; *n* (%)	9 (23%)	21 (32%)	0.3380

*BMI* body mass index, *HCM* hypertrophic cardiomyopathy, *LVEF* left ventricular ejection fraction, *LVWT* left ventricular wall thickness, *LVOT* left ventricular outflow tract, *SCD* sudden cardiac death, *VUS* variant of uncertain significance.

### STRUM exhibits improved specificity over established sequence-based prediction algorithms and improved sensitivity when combined with CardioBoost

**Fig. 3:**
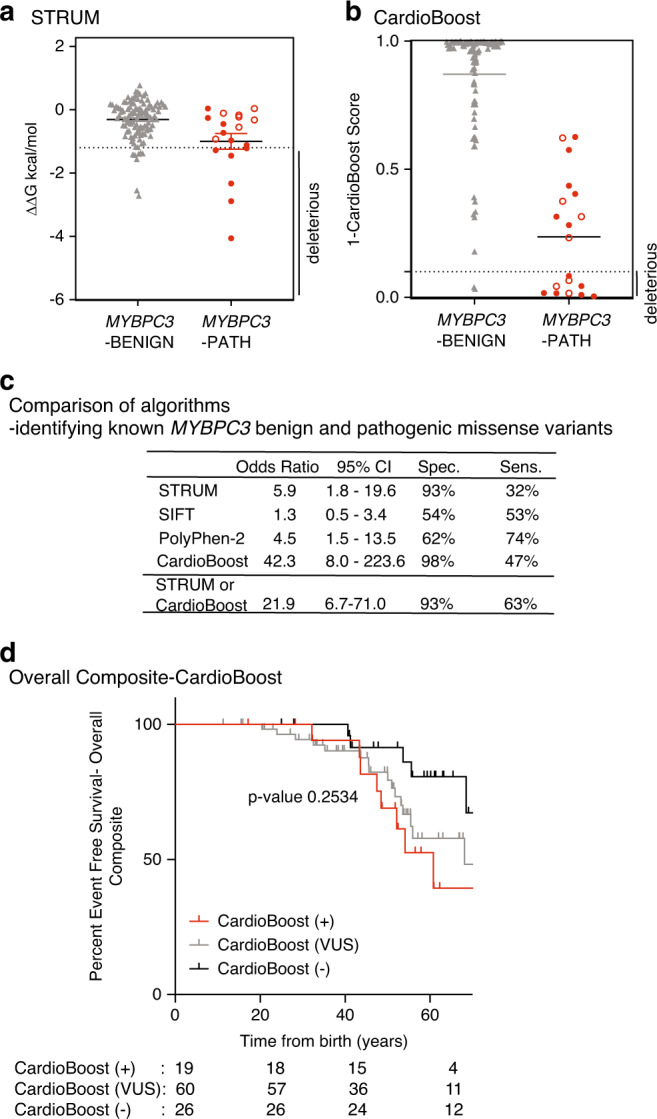
STRUM is complementary to CardioBoost. **Results of computational analysis for each unique** ***MYBPC3*-Benign (gray triangles,** ***n*** = **110) and** ***MYBPC3*-Path (red circles,** ***n*** = 19) variant. (**a**) STRUM. (**b**) CardioBoost. Mean and SEM for each group depicted. The cutoff for deleterious variants for STRUM was ΔΔG ≤ −1.2 kcal/mol. The cutoff for deleterious variants for CardioBoost (CardioBoost +) was a probability score > 0.90; this is graphed as 1-CardioBoostScore < 0.10. C3 pathogenic variants are depicted in open circles in (**a**) and (**b**). (**c**) Statistical analysis of computational method utilized here in STRUM (Fig. 3), CardioBoost (Fig. 3), SIFT (Figure S6), and PolyPhen-2 (Figure S6) is shown including odds ratio, 95% confidence interval (CI), sensitivity, and specificity. (**d**) Using the same patient selection criteria in the Sarcomeric Human Cardiomyopathy Registry (SHaRe) detailed in Fig. 1, patients with hypertrophic cardiomyopathy (HCM) and a MYBPC3 missense variants of uncertain significance (VUS) were analyzed by CardioBoost. CardioBoost (+) was a probability score > 0.90, CardioBoost VUS ≥ 0.10 and ≤0.90, and CardioBoost (−) < 0.10. Of the 105 patients analyzed by STRUM 19 were CardioBoost (+). Kaplan–Meier curves reveal that patients carrying a CardioBoost (+) *MYBPC3* VUS (red) exhibited higher rates of adverse HCM-related outcomes (overall composite) than patients carrying a CardioBoost (−) MYBPC3 VUS (black); however, the null hypothesis could not be excluded, *p* value 0.0945. This remains true when comparing patients carrying a MYBPC3 VUS that is CardioBoost (+) (red), CardioBoost (VUS) (gray), and CardioBoost (−) (black) (*p* value 0.2534).

To determine the sensitivity and specificity of STRUM to differentiate pathogenic from benign variants within *MYBPC3* we performed STRUM analysis on all known pathogenic missense variants within ShaRe (*n* = 19) and known missense benign variants within SHaRe and gnomAD (*n* = 110, Table S1, Fig. 3a). These variants were present in 412 patients with HCM within the SHaRe registry. *MYBPC3* benign variants exhibited a mean ΔΔG of −0.31 ± 0.60 kcal/mol, which was significantly higher than *MYBPC3* VUS (ΔΔG of −0.73 ± 1.06 kcal/mol, *p* = 0.005) (Figure S4) and *MYBPC3* pathogenic variants (mean ΔΔG of −1.00 ± 1.08 kcal/mol, *p* = 0.016) (Fig. 3a). We found that variants predicted to be deleterious by STRUM were more likely to be pathogenic variants (odds ratio [OR] 5.9, 95% CI 1.8–19.6) (Fig. 3c). Only nine additional unique nontruncating *MYBPC3* variants were designated as pathogenic and/or likely pathogenic within ClinVar. However, all of these variants had a single submission and a review status of 0–1/4 criteria provided. By modern standards, these variants would be reclassified as VUS and were therefore not included in our analysis.

Algorithms that were purely sequence-based achieved greater sensitivity but performed inferiorly to STRUM in regard to specificity. STRUM exhibited a 93% specificity for benign variants and PolyPhen-2 and SIFT exhibited a specificity of 62% (OR 4.5, 95% 1.5–13.5) and 54% (OR 1.3, 95% CI 0.5–3.4) respectively (Fig. 3c, Figure S6). Additionally, variant interpretation by SIFT or PolyPhen-2 did not stratify patients carrying a *MYBPC3* VUS for clinical adverse outcomes (Figure S6).

In comparison, CardioBoost demonstrated a specificity of 98% (OR 42.3, CI 8.0–223.6) (Fig. 3, Table S1). For pathogenic variants, CardioBoost demonstrated a sensitivity of 47%. Interestingly, there was limited overlap among known pathogenic variants predicted to be deleterious by STRUM and those predicted to be deleterious by CardioBoost, making the two algorithms complementary (Table S1). Combining these algorithms to classify any variant predicted to be deleterious by CardioBoost or STRUM as pathogenic maintained a high specificity of 93% and improved sensitivity to 63% (Fig. 3c).

When examining patients with HCM and a *MYBPC3* missense VUS, STRUM identified a larger number of *MYBPC3* VUS as deleterious. Only 16 of 39 (41%) patients with a STRUM+ *MYBPC3* VUS were also identified as CardioBoost+. Just three additional patients were uniquely identified as CardioBoost+ (Table S2). While there is a trend toward a higher rate of adverse clinical events in patients with HCM and a CardioBoost+ *MYBPC3* VUS, this difference was not statistically significant (Fig. 3d).

### STRUM predictions within pathogenic variants are consistent with experimental modeling

**Fig. 4:**
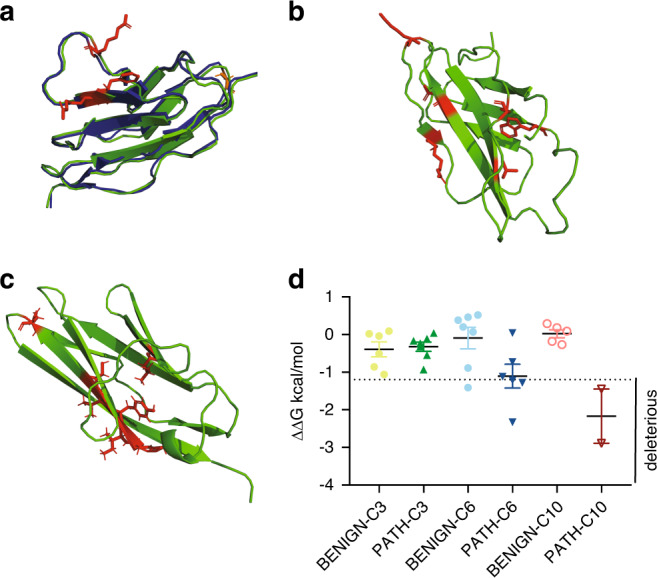
Structural analysis of pathogenic missense *MYBPC3* variants. MyBP-C (the protein encoded by *MYBPC3*) domains C3, C6, and C10 were structurally modeled using I-TASSER^33–35^ (PyMOL,cartoon, green). Wild-type residues that are affected by missense pathogenic variants are depicted in red (PyMOL, sticks). (**a**) For the C3 domain, the I-TASSER model is aligned with an available NMR structure (2mq0.pdb,^28^ blue, PyMOL cartoon). Pathogenic variants within C3 largely cluster in a surface-exposed region. (**b**) C6 domain and (**c**) C10 domain pathogenic variants do not cluster within a specific region of the domain. (**d**) Results of STRUM^19^ analysis for *MYBPC3* pathogenic and benign variants within C3, C6, and C10 are shown, with mean and SEM for each group depicted. Graph is labeled to indicate variants predicted to be deleterious.

Prior experimental characterization of *MYBPC3* pathogenic missense variants within the C10 domain, Leu1238Pro and Asn1257Lys, demonstrated that these variants failed to localize to the sarcomere and were rapidly degraded within primary cardiomyocytes.^14^ Consistent with these experimental findings, pathogenic C10 domain variants are uniformly predicted to destabilize protein folding (ΔΔG of −2.89 and −1.45 kcal/mol respectively) (Fig. 4).

Conversely, of the pathogenic *MYBPC*3 variants not predicted to be deleterious by STRUM (Fig. 3), a large number were localized within the C3 domain (Fig. 3a, open circles; 7/13) and exhibited a mean ΔΔG −0.32 kcal/mol, (range −0.93 to 0.04). A large number of known pathogenic variants cluster within the C3 domain near a surface-exposed flexible linker (Fig. 4).^15^ Thus, these variants would be predicted to alter electrostatic protein–protein interactions but would not be expected to disrupt subdomain folding. This result is consistent with prior experimental and structural characterization data of these C3 pathogenic variants. Arg495Gln, Arg502Trp, and Phe503Leu incorporate normally into the sarcomere and have protein half lives that are indistinguishable from wild-type MyBP-C within primary cardiomyocytes.^14^ Further, the NMR structure of the *MYBPC3* Arg502Trp C3 domain reveals preserved subdomain folding.^28^

While C3 and C10 pathogenic variants have a narrow range of ΔΔG values, ΔΔG predictions for C6 pathogenic variants vary from −2.33 to 0.04 (mean ΔΔG −1.11). We previously examined two C6 domain variants, Arg810His and Trp792Arg, and found that they incorporate normally into the sarcomere and exhibit normal protein half lives in primary cardiomyocytes.^14^ However, both of these variants were predicted to destabilize subdomain folding by STRUM, exhibiting values near the cutoff: Arg810His (ΔΔG −1.22 kcal/mol), Trp792Arg (ΔΔG −1.28 kcal/mol). They are also predicted to be pathogenic by CardioBoost (Table S2). These observations suggest that a subset of pathogenic variants mildly disrupt subdomain folding without causing complete destabilization of MyBP-C. Subdomain destabilization in these cases could interfere with protein–protein interactions or MyBP-C conformational dynamics.

### In silico saturation mutagenesis of *MYBPC3* identified 4,943 missense variants predicted to cause subdomain misfolding

Only a subset of amino acid substitutions has been observed in patients with HCM and are cataloged in publicly available databases, such as ClinVar. However, previously unreported variants frequently arise in probands with HCM who undergo clinical genetic testing.^29^ Thus, we performed STRUM on all possible *MYBPC3* single amino acid substitutions (in silico mutagenesis) to develop a compendium of STRUM+ variants that may be useful for the research and clinical community. We found that 4,943 of 24,665 (20%) amino acid substitutions were predicted to disrupt subdomain folding (Figure S6, Tables S4, S5).

## DISCUSSION

Clinical risk stratification has been a cornerstone of clinical HCM management. It is well-established that patients with sarcomeric HCM have a higher rate of adverse clinical outcomes compared with nonsarcomeric HCM, enabling the incorporation of genetic data into clinical risk stratification in HCM.^8,18^ Yet, refinement of clinical risk for patients with a VUS remains an ongoing challenge for clinicians.^1,5^ We have identified a subpopulation of patients with a *MYBPC3* missense VUS that are predicted to disrupt subdomain protein folding (STRUM+) who exhibit clinical outcomes indistinguishable from patients with a pathogenic *MYBPC3* variant. Conversely, patients carrying a *MYBPC3* VUS not predicted to affect subdomain folding (STRUM−), exhibit a lower prevalence of adverse clinical outcomes similar to patients with nonsarcomeric HCM. Although the methodology of parsing these variants is different for *MYBPC3* because of differing underlying mechanisms, these findings are analogous to a recent study in *MYH7* in which patients with HCM carrying VUS that were located within the interacting heads motif had a higher rate of adverse clinical outcomes compared with patients carrying VUS that were outside of this motif.^30^ These studies together suggest that VUS in sarcomere genes are primarily an admixture of pathogenic and benign variants. So, while patients with HCM carrying sarcomere gene VUS as a whole exhibit a prevalence of clinical outcomes that are intermediate between patients with or without pathogenic sarcomere variants,^8^ a computational approach specifically leveraging the pathogenic mechanism of *MYBPC3* has enabled the identification of higher risk subpopulation that exhibit clinical outcomes similar to sarcomeric HCM and a lower risk subpopulation that exhibit clinical outcomes similar to nonsarcomeric HCM.

While computational prediction should not be exclusively relied on to assign pathogenicity of a variant or risk stratify an individual patient, STRUM could be incorporated in an additive manner with other methods for variant adjudication to prioritize variants that warrant further investigation. Given that novel *MYBPC3* variants are frequently identified by genetic testing of probands with HCM,^29^ we completed an in silico “saturation mutagenesis” of *MYBPC3* compiling a complete list of STRUM+ variants. Excluding known pathogenic or benign variants, we estimate that ~0.097% (1/1,033) individuals within gnomAD carry a *MYBPC3* variant predicted to cause subdomain misfolding by STRUM. STRUM+ *MYBPC3* VUS identified in patients with HCM should be prioritized for additional clinical and experimental investigation. Specifically, functional experimental studies to evaluate the direct effects of *MYBPC*3 VUS on protein stability, folding, and localization, as we have done previously for a subset of pathogenic variants,^14^ will be important. Familial cosegregation analysis on patients carrying a *MYBPC3* STRUM+ VUS would add complementary information to these types of experimental studies.

When known benign missense variants were evaluated by STRUM, 102 of 110 variants were correctly predicted, with an overall specificity of 93%. However, for known pathogenic variants, only 7 of 19 were predicted to alter subdomain folding by STRUM, yielding a sensitivity of 32%. This lower sensitivity was in large part explained by a known cluster of pathogenic variants within C3.^15^ None of the seven known pathogenic variants in C3 had a ΔΔG value below the threshold of −1.2 kcal/mol. This is consistent with experimental data that demonstrate C3 variants localize normally to the sarcomere and exhibit protein half lives similar to wild-type MyBP-C. Additionally, an NMR structure of Arg502Trp demonstrates that this variant does not disrupt subdomain folding but rather is more likely to alter protein–protein interactions.^14,28^ In contrast, MyBP-C pathogenic variants in C10, predicted by STRUM to cause subdomain misfolding, fail to localize to the sarcomere and are rapidly degraded.^14^ These experimental results support the accuracy of STRUM predictions for subdomain misfolding. Further, they highlight that STRUM is only predictive of pathogenicity for variants that significantly alter protein folding as their primary mechanism. Thus, a ΔΔG value of > −1.2 kcal/mol does not exclude pathogenicity for variants that cause loss or gain-of-function through an alternate mechanism such as alternative splicing or altered protein–protein interactions. STRUM is best applied to VUS after other clinical, computational, and experimental criteria for variant adjudication have been implemented. For example, *MYBPC3* pathogenic variants that lead to LoF by mechanisms other than subdomain misfolding have previously been well characterized and defined as pathogenic, including splice variants^14,22,23^ and the cluster of pathogenic variants within C3 (aa.485–503)^15,28,31^ discussed above.

STRUM performed superiorly to sequence based algorithms alone, such as SIFT and PolyPhen-2, which each had lower specificity and were unable to clinically risk stratify patients with HCM and a *MYBPC3* missense VUS. Compared with using each method independently, combining STRUM and CardioBoost improved sensitivity for identifying known pathogenic variants to 63% while maintaining a specificity for known benign variants of 93%. CardioBoost supported pathogenicity for four missense VUS that were STRUM - but only predicted pathogenicity for 12/34 of *MYBPC3* STRUM+ VUS. This result highlights the added utility of STRUM to identify a subset of VUS within *MYBPC3* that result in local subdomain misfolding leading to allelic LoF and have a high probability of being pathogenic. Because CardioBoost and STRUM are complementary and have high specificity, we would propose that the ACMG/AMP PP3 criteria, where multiple lines of computational evidence support a deleterious effect of a variant, could be applied when one or both algorithms predict pathogenicity. Conversely, because of relatively limited sensitivity for each algorithm independently, we would propose that the BP4 criteria, where multiple lines of computational evidence support no impact of the variant, be applied only if both algorithms predict that a variant is nonpathogenic.

Although this study was limited by a moderate sample size of 105 patients with HCM, the comprehensive variant adjudication in SHaRe enabled strict inclusion of patients carrying a single VUS within *MYBPC3* to clearly discriminate genetic–clinical correlates in this population. This approach enabled us to discern a difference in a composite of adverse clinical outcomes between patients with STRUM+ and STRUM- variants. However, the sample size was insufficient for detecting differences in individual outcomes, such as arrhythmias or heart failure, and did not provide sufficient power to correct for other risk predictors.

The approach of using STRUM as an adjunctive tool for decision making may also be applicable to other genes for which LoF is a pathogenic mechanism. Approximately 50% of disease associated variants within Human Gene Mutation Database are truncating variants predicted to result in LoF.^11^ These genes, like *MYBPC3*, also have missense VUS that may be evaluated for protein misfolding using STRUM. For example, there are several causal genes for hypertrophic, dilated, and arrhythmogenic cardiomyopathies with truncating pathogenic variants, including lamin A/C, desmoplakin, and plakophilin 2, Titin, and phospholamban.^11,32^ This approach is best suited for nonenzymatic proteins where high-quality structural modeling can be performed, and for which the primary pathogenic mechanism has been established to be LoF.

### Conclusions

We show that the computational algorithm STRUM, that predicts protein structure stability in response to missense variation, enables identification of patients carrying a *MYBPC3* VUS who may be at higher clinical risk of adverse events. This approach also provides supportive evidence for pathogenicity, prioritizing variants for functional experimental studies and clinical familial segregation to improve *MYBPC3* variant adjudication. Finally, STRUM may be broadly applicable to variants in other genes for which LoF is an established mechanism.

## Supplementary information

Supplementary Information

Supplementary Table S1

Supplementary Table S2

Supplementary Table S5

## Data Availability

De-identified data will be made available by request to the authors.
