# Supplementary material for: Computational prediction of protein subdomain stability in MYBPC3 enables clinical risk stratification in hypertrophic cardiomyopathy and enhances variant interpretation
Source: Genet Med. 2021 Mar 29;23(7):1281–7. doi: 10.1038/s41436-021-01134-9 (PMC8257482; doi:10.1038/s41436-021-01134-9)
Supplement: Supplementary file 1 — Supplementary Information [file 41436_2021_1134_MOESM1_ESM.pdf]

Supplemental Information: Computational prediction of protein subdomain stability in *MYBPC3* enables clinical risk stratification in hypertrophic cardiomyopathy and enhances variant interpretation.

Authors: Andrea D. Thompson, MD, PhD; Adam S. Helms, MD, MS, Anamika Kannan; Jaime Yob; Neal K. Lakdawala, MD; Samuel G. Wittekind, MD, MSc; Alexandre C. Pereira, MD, PhD; Daniel L. Jacoby, MD; Steven D. Colan, MD; Euan A. Ashley, MRCP, Dphil; Sara Saberi, MD, MS; James S. Ware, PhD, MRCP; Jodie Ingles, PhD; Christopher Semsarian, MBBS, PhD, MPH; Michelle Michels, MD, PhD; Iacopo Olivotto, MD; Carolyn Y. Ho, MD; Sharlene M. Day, MD; SHaRe investigators.

Table of Contents

A. Expanded Methods **3-8**

B. Figures **9-17**

Figure S1: MyBP-C subdomain I-TASSER models 9

Figure S2: I-TASSER Models of proline rich (PR) and M-domain linkers 10

Figure S3: Structural model of MyBP-C 11-12

Figure S4: Computational analysis of missense *MYBPC3* VUSs 13-14

Figure S5: Risk stratification of individual composite outcomes. 15

Figure S6: Sequence-based algorithms were exhibit decreased specificity compared to STRUM and are unable to identify a subgroup of HCM patients with a *MYBPC3* VUS at increased risk for HCM-related adverse clinical outcomes. 16-17

Figures S7: STRUM Analysis of *MYBPC3* VUSs 18

C. Tables **19-23**

TableS1: Computation analysis of *MYBPC3* non-synonymous missense variants- Benign and Path 19

Table S2: Computation analysis of *MYBPC3* non-synonymous missense variants- VUS within SHaRe 20

Table S3: Summary of STRUM inputs and resulting I-TASSER models 21

Table S4: STRUM results by subdomain 22

Table S5: Complete STRUM analysis of *MYBPC*3 Variants 23

D. Supplemental References **24-27**

Expanded Methods

A. Variant Interpretation

SHaRe non-truncating *MYBPC3* missense variants (Supplemental Tables 1,2) were classified as previously reported[1] in accordance with American College of Medical Genetics and Genomics (ACMGG) guidelines, leveraging available clinical and experimental data[1-5]. Herein we provide additional detail regarding this variant interpretation process. Clinically-indicated genetic testing was performed at all sites. Initial site designations of Pathogenic/Likely Pathogenic (pathogenic), variant of unknown significance (VUS), benign/likely benign (benign) were reviewed for accuracy in accordance with American College of Medical Genetics and Genomics (ACMGG) guidelines[6, 7] This review included review of available clinical and experimental data such as variant frequency (ShaRe and gnomAD) and evidence of segregation within families either previously published or within SHaRe. Further, splice site variants were considered pathogenic if they affected critically conserved splice consensus sites (i.e. acceptor -1 or -2; donor +1 or +2) and/or if experimental evidence showed aberrant splicing[1, 3, 5, 6]. All potential exonic splice variants were identified and were cross-referenced to recent *in silico* and mini-gene assay analysis of MYBPC3 variants predicted to impact splicing[1, 3, 5, 6]. None of the variants included in this study had experimental evidence of splicing, as variants with proven evidence of splicing were classified as truncating. Variants were considered "benign/likely benign" if the population allele frequency exceeded 0.004 and the odds ratio between SHaRe and gnomAD was <10-fold over the gnomAD allele frequency. If the variant was absent in gnomAD a conservative upper bound population allele frequency of 4.5E-06 was used for the odds ratio calculation[1]. Since variants in sarcomere gene present in gnomAD with allele frequencies of > 4E-05 and absent in SHaRe are unlikely to be independently pathogenic for HCM, these variants were included in our list of benign MYBPC3 variants[1].

We also reference previously established subgroups within the SHaRe registry Sarc+, SarcU, Sarc-[8-10]. Sarc+ is defined as sarcomeric HCM or patients with HCM carrying a pathogenic or likely pathogenic variant within 8 sarcomere genes definitively associated with HCM (MYBPC3, MYH7, TNNT2, TNNI3,

TPM1, MLY2, MYL3, ACTC)[8]. SarcU (also referred to as SarcVUS in previous publications) is defined as HCM patients carrying a sarcomere variant of uncertain significant(s) and Sarc- is defined as non-sarcomeric HCM or patients with HCM who underwent genetic testing and had no pathogenic, likely pathogenic, or VUS variant identified within a sarcomere gene. Patients were excluded from Sarc- if they had pathogenic variants in genes encoding non-sarcomere proteins such as GLA or LAMP2.

B. Computational Protein Folding Stability Predictive Modeling

For *MYBPC3*, which encodes protein MyBP-C, missense variants were analyzed using STRUM. STRUM calculates the effect of the missense variant on the Gibbs free energy of local subdomain folding (referred to as the ΔΔG, kcal/mol)[11]. Subdomains were selected instead of the full-length protein to enable more accurate structural modeling (Supplemental Table 3). STRUM analysis of *MYBPC3* variants was performed using amino acid sequence inputs (Supplemental Table 3) and method 2, completed 05/2020. This enabled a full analysis of all possible MYBPC3 subdomain variants (Supplemental Table 6).

A negative ΔΔG value indicates the degree of reduced folding energy (kcal/mol) relative to the wild-type subdomain, or folding destabilization[11]. Previous experimental validation of this algorithm*f* compared STRUM predictions to 3421 experimentally tested variants from 150 proteins and demonstrated a strong correlation with a Pearson's correlation coefficient (PCC) of 0.79 and root mean square error (RMSE) of prediction of 1.2 kcal/mol[11]. A value of ΔΔG ≤-1.2 kcal/mol was defined as the cut-off for destabilizing (deleterious) variants while a value of ΔΔG > -1.2 was defined as the cut-off for variants that did not significant destabilize subdomain folding (non-deleterious) variants.

C. Computational Structural Modeling Information

The STRUM algorithm utilizes I-TASSER structural models in calculating ΔΔG. I-TASSER (Iterative Threading ASSEmbly Refinement) is a hierarchical approach to protein structure and function prediction[12-14]. MyBP-C is made up of immunoglobulin and fibronectin subdomains[15]. Structural predictions of MyBP-C (the protein encoded by *MYBPC3*) subdomains C0-C10 were obtained using I-

TASSER and are depicted using PyMOL (Supplemental Figure 1). Two linker regions were modeled. The first is a proline rich region between C0 and C1. The second is the M-domain linker between C1 and C2. For variants within these linker regions, a structural model of two adjacent subdomains and the linker region of interest were established (Supplemental Figure 2). Linker regions were otherwise assumed to consistent of short flexible unstructured linkers and were not analyzed using STRUM (Supplemental Figure 3, Supplemental Table 4).

High quality I-TASSER models were produced for each sub-domain (Supplemental Figure 1-3, Supplemental Table 3)[12, 13]. To describe quality metrics in more detail; the C-score is a confidence score for estimating the quality of predicted models by I-TASSER It is calculated based on the significance of threading template alignments and the convergence of parameters of the structure assembly simulations. C-score is typically in the range of [-5,2], where a C-score of higher value signifies a model with high confidence. Subdomains C0-C10 exhibited C-scores ranging from 0.22-0.98. Another measure of model quality is TM-score, which is sensitive to local error. A TM-score > 0.5 indicates a model with correct topology. Our models exhibited TM-scores ranging from 0.74-0.84. Finally, we report a root mean square deviation (RMSD) compared to known MyBP-C subdomain structures, which ranged from 0.29-0.44 Angstroms[16-23], exhibiting good agreement between experimental and modeling data.

With the exception of two linker regions, individual subdomains are connected by short flexible linkers which were not modeled (Supplemental Figure 3). The two larger linker regions present in MyBP-C are; the proline rich region between C0 and C1 domains and the M-domain between C1 and C2 domains[15]. These linker regions were modeled in the presence of their two adjacent subdomains; C0-Proline rich linker-C2 (aa 1-260) and C1-M domain-C2 (aa 152-452) using I-TASSER (Supplemental Figure 2). This revealed a flexible proline rich linker with minimal secondary structure and a structured M-domain made up of beta-sheets. The M-domain model is distinct from a truncated region of M-domain that was evaluated connected to C2 domain by NMR (319-451) which suggested a triple helix within this C-terminal portion of the M-domain (PDB 5k6p.pdb)[21]. These models were of acceptable quality with C-score of -2.01 and -0.72 and TM score 0.47 and 0.62 respectively, albeit lower quality than individual

subdomains. As such these models were only utilized to evaluate variants within the linker region. All depictions of I-TASSER models and alignments with known MyBP-C structures were performed using PyMOL (Supplemental Figure 1-3).

Finally, a model of full length *MYBPC3* based on axial-radial model[24] using these individual subdomains was assembled (Supplemental Figure 3). This model serves to illustrate the well- defined subdomain immunoglobulin and fibronectin folds within *MYBPC3* and assumes independent motion of individual subdomains connected by flexible linker regions. The exceptions to this assumption are the proline-rich (PR) linker between CO and C1 and the M-domain linker between C1 and C2 which were modeled as described above. There are multiple proposed models of MyBP-C quaternary structure within the sarcomere based on electron microscopy data[24-27]. Further experimental work is needed to more accurately model orientation between subdomains, potential interdomain interactions, and *MYBPC3* quaternary structure.

D. Clinical Outcomes Analysis

Only HCM patients with a single *MYBPC3* variant were included in clinical outcomes analysis to avoid confounding from multiple cases with multiple sarcomere gene variants (Figure 3). To expand on this inclusion criterion further, we began by identifying 426 SarcU patients within SHaRe. As previously described[8] SarcU patients are HCM patients whom have undergone clinical genetic testing and lack pathogenic or likely pathogenic variants within sarcomere genes or other potentially pathogenic genes such as GLA or LAMP2 but do carry a VUS within a sarcomere gene. We next limited our analysis to patients with a single VUS [VUS variants (all genes): one], excluding patients that carry multiple sarcomere VUSs. Finally, we limited our analysis to patients carrying a single missense MYBPC3 VUS [VUS variants (MYBPC3): STRUM] (Figure 3). For comparison groups (Figure 4) we evaluated HCM patients with a single MYBPC3 pathogenic variant (MYBPC3-Path-all). To identify this group we utilized the previously defined Sarc+ group[8], limiting our evaluation to patients with single pathogenic

sarcomere variant (P/LP variants: One) and excluding patients carrying another VUS (VUS variants: zero). Our final comparison group was the previously defined Sarc- group[8].

E. Defining Clinical outcomes

Composite clinical outcomes evaluated herein as previously defined[8] are;

- Ventricular arrhythmic composite: first occurrence of sudden cardiac death, resuscitated cardiac arrest, or appropriate implantable cardioverter-defibrillator therapy
- Heart Failure composite: first occurrence of cardiac transplantation, LV assist device implantation, LV ejection fraction <35%, or New York Heart Association class III/IV symptoms
- Overall composite: first occurrence of any component of the ventricular arrhythmic or heart failure composite end point (without inclusion of LV ejection fraction), all- cause mortality, atrial fibrillation (AF), stroke, or death

F. Statistical Analysis

Odds ratio(OR) , sensitivity, and specificity were calculated to evaluate the association between computational algorithms (STRUM, SIFT, PolyPhen-2) STRUM predictions of deleterious variants and known *MYBPC3* pathogenic and benign variants as follows;

$$OR = (A \times D) \div (B \times C)$$

$$Specificty = D \div (B + D)$$

$$Sensitivity = A \div (A + \mathrm{C})$$

$$95\%\ CI = \exp[\ln(OR) - 1.96 \times SE\{\ln(OR)\}]\ to\ \exp[\ln(OR) + 1.96 \times SE\{\ln(OR)\}]$$

where

A = variants predicted the be deleterious and known to be pathogenic,

B = variants predicted to be deleterious and known to be benign,

C= variants predicted to be non-deleterious and known to be pathogenic,

D= variants predicted to be non-deleterious and known to be benign.

$$SE\{\ln(OR\} = \sqrt{(\frac{1}{A} + \frac{1}{B} + \frac{1}{C} + \frac{1}{D})}$$

Missense *MYBPC3*		
	MYBPC3-Path	*MYBPC3*-Benign
STRUM+	6	7
STRUM -	13	103
	MYBPC3-Path	*MYBPC*3-Benign
SIFT+	10	51
SIFT -	9	59
	MYBPC3-Path	*MYBPC3*-Benign
PolyPhen-2+	14	42
PolyPhen-2 -	5	68
	MYBPC3-Path	*MYBPC3*-Benign
CardioBoost+	9	2
CardioBoost-	10	94
	MYBPC3-Path	*MYBPC3*-Benign
STRUM+ OR CardioBoost+	12	8
STRUM- AND CardioBoost-	7	102

G. Figures

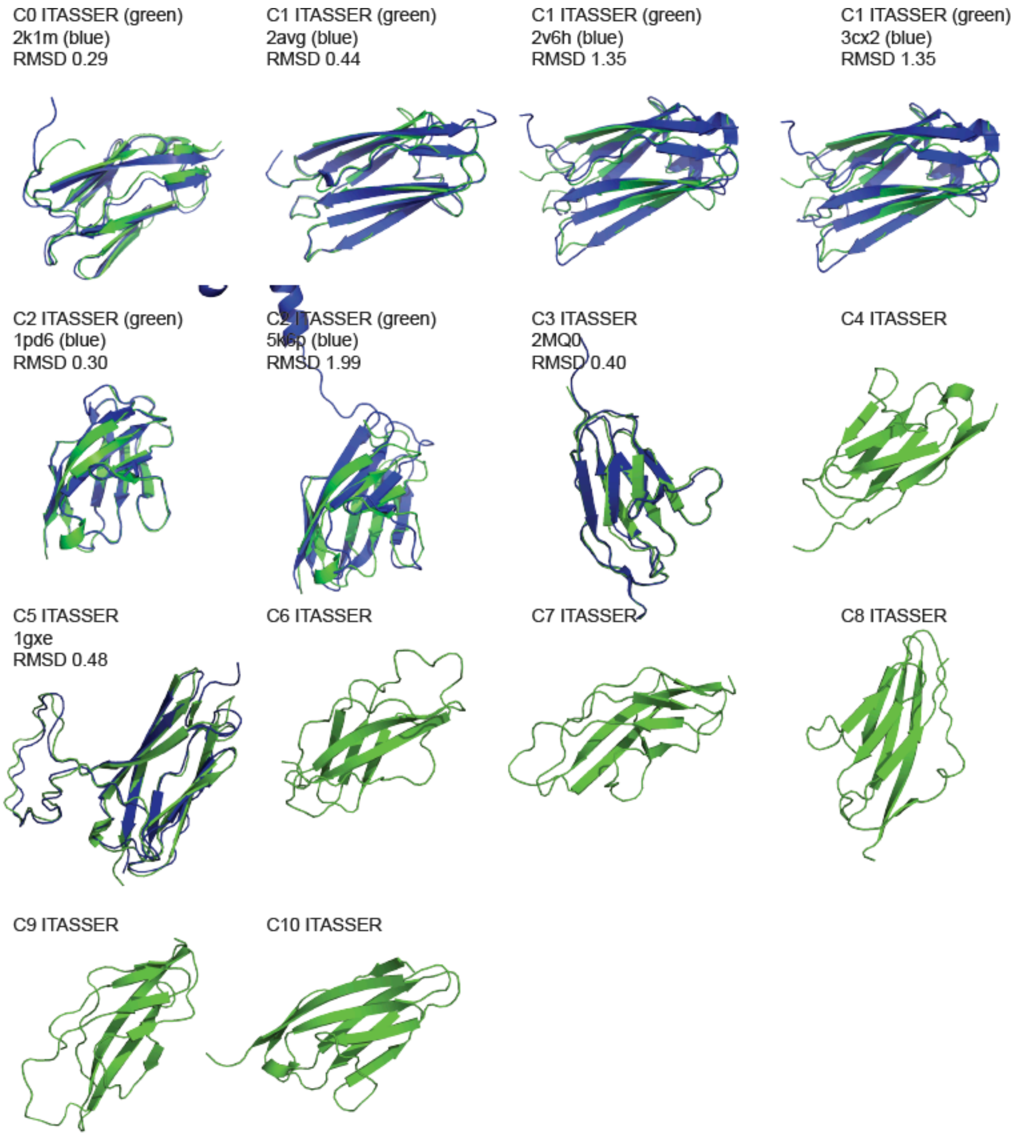

Figure S1: MyBP-C subdomain I-TASSER models. I-TASSER models were derived for MyBP-C subdomains C0-C10 from sequence inputs as detailed in Supplemental Table 3 are shown in green (PyMOL, cartoon). These structures were aligned with all available known MyBP-C subdomain structures shown in blue (PyMOL, cartoon)[16-23]. For each alignment the pdb code of the experimentally derived structure and the root mean square deviation (RMSD) of the alignment is reported. Subdomains C4, C6, C7, C8, C9, C10 did not have available structural data for comparison.

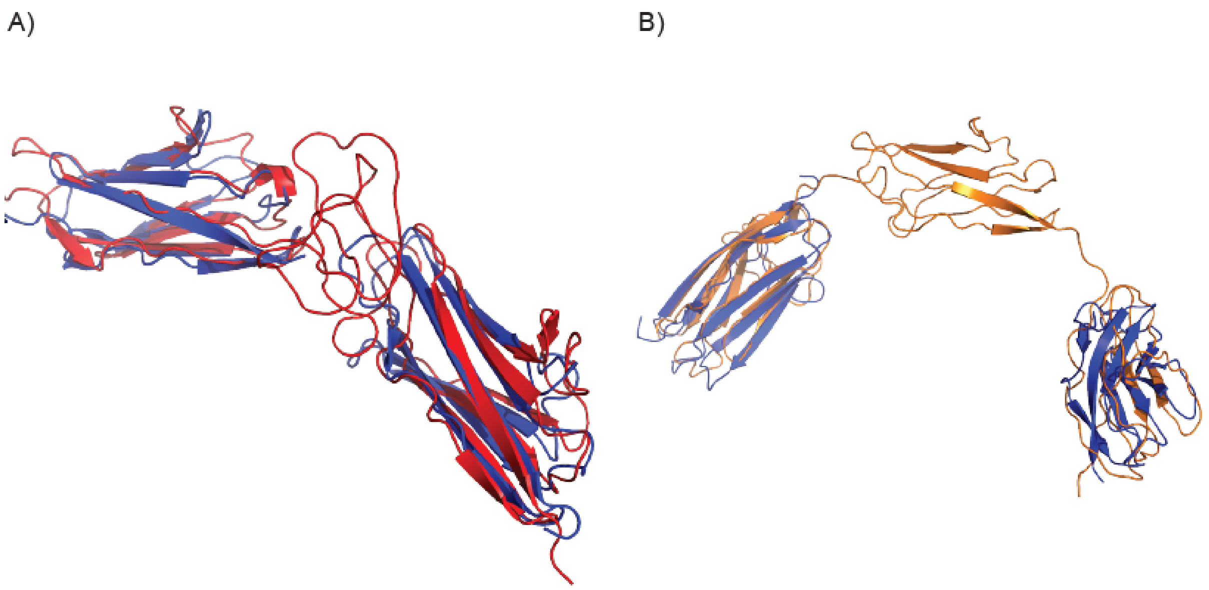

Figure S2: I-TASSER Models of proline rich and M-domain linkers. I-TASSER models of these two linker regions were made by modeling the linker regions with two adjacent domains. A) The proline rich (PR) linker model [C0-PR-C1 (aa 1-260)] is shown in red (PyMOL, cartoon). This model was aligned with known MyBP-C C0 and C1 subdomain structures shown in blue (PyMOL, cartoon) (2k1m.pdb and 2avg.pdb respectively). B) The M-domain linker model [C1-M domain-C2 (aa 152-452)] is shown in orange (PyMOL, cartoon). This model was aligned with known MyBP-C C1 and C2 subdomain structures shown in blue (PyMOL, cartoon). (2avg.pdb, and 1pd6.pdb respectively) [16, 17]

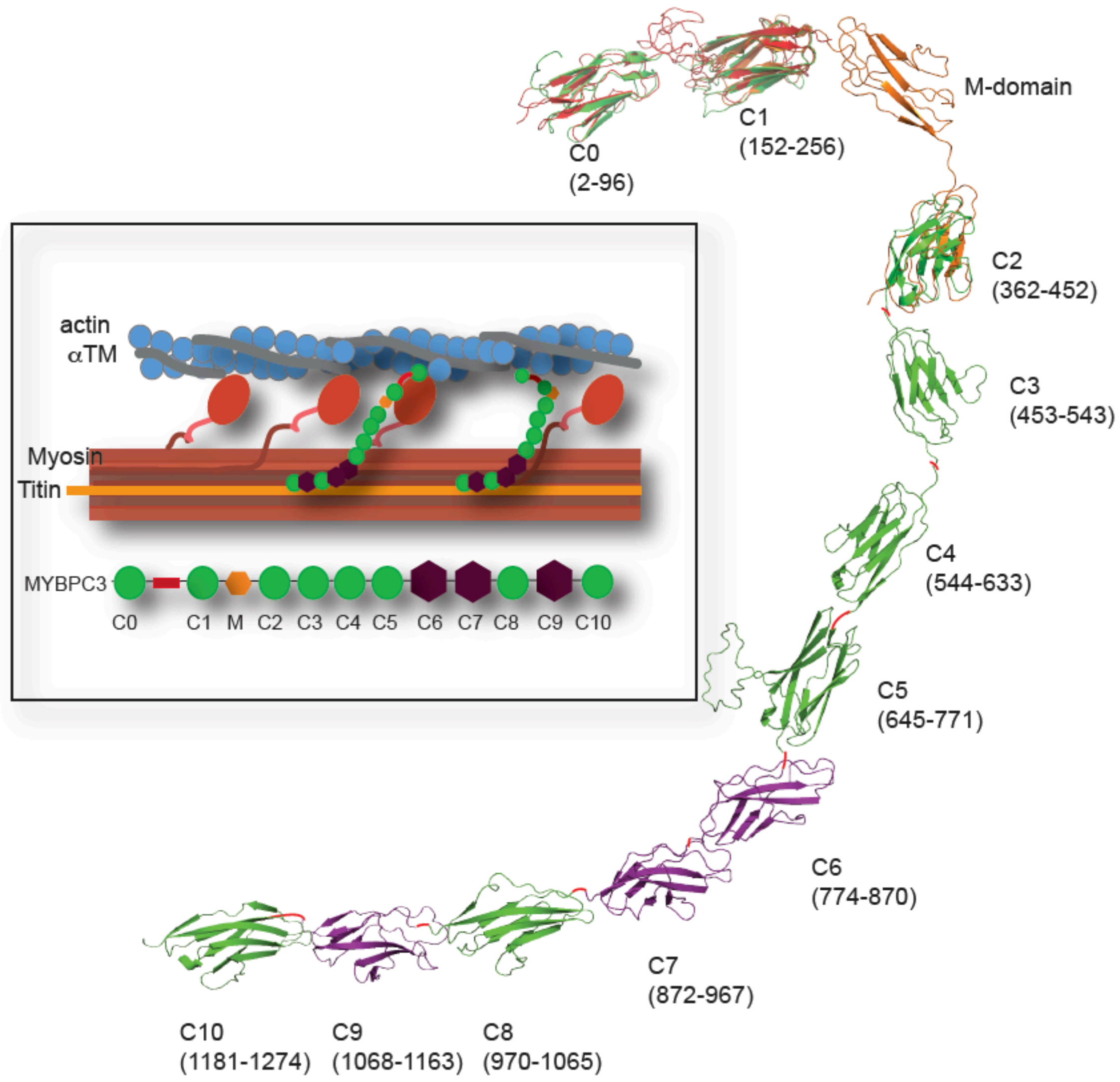

Figure S3: Structural model of MyBP-C. MyBP-C (the protein encoded by *MYBPC3*) is made up of immunoglobin (green circles) and fibronectin domains (purple hexagons) connected by short flexible linkers. Two larger linker regions exist; the proline-rich (PR) linker (red rectangle) between C0 and C1 domains and the M-domain linker (small orange hexagon) between C1 and C2 domains. MyBP-C is positioned in an anti-parallel fashion within the A-band of sarcomere. The N-terminus (C0-C2) interacts with actin and myosin in a dynamic fashion while the C-terminus (C7-C10) interacts with thick filament and Titin. A structural model was built using I-TASSER subdomain models. Individual immunoglobulin (green PyMOL cartoon) and fibronectin (purple PyMOL cartoon) I-TASSER models are arranged based on previously proposed quaternary structural model[24]. In addition, the C0- PR-C1 (red, PyMOL cartoon)

and C1-Mdomain-C2 (orange PyMOL cartoon) I-TASSER models were aligned with the individual I-TASSER models for these subdomains. This model otherwise assumes independent motion of individual subdomains connected by flexible linker regions (red lines).

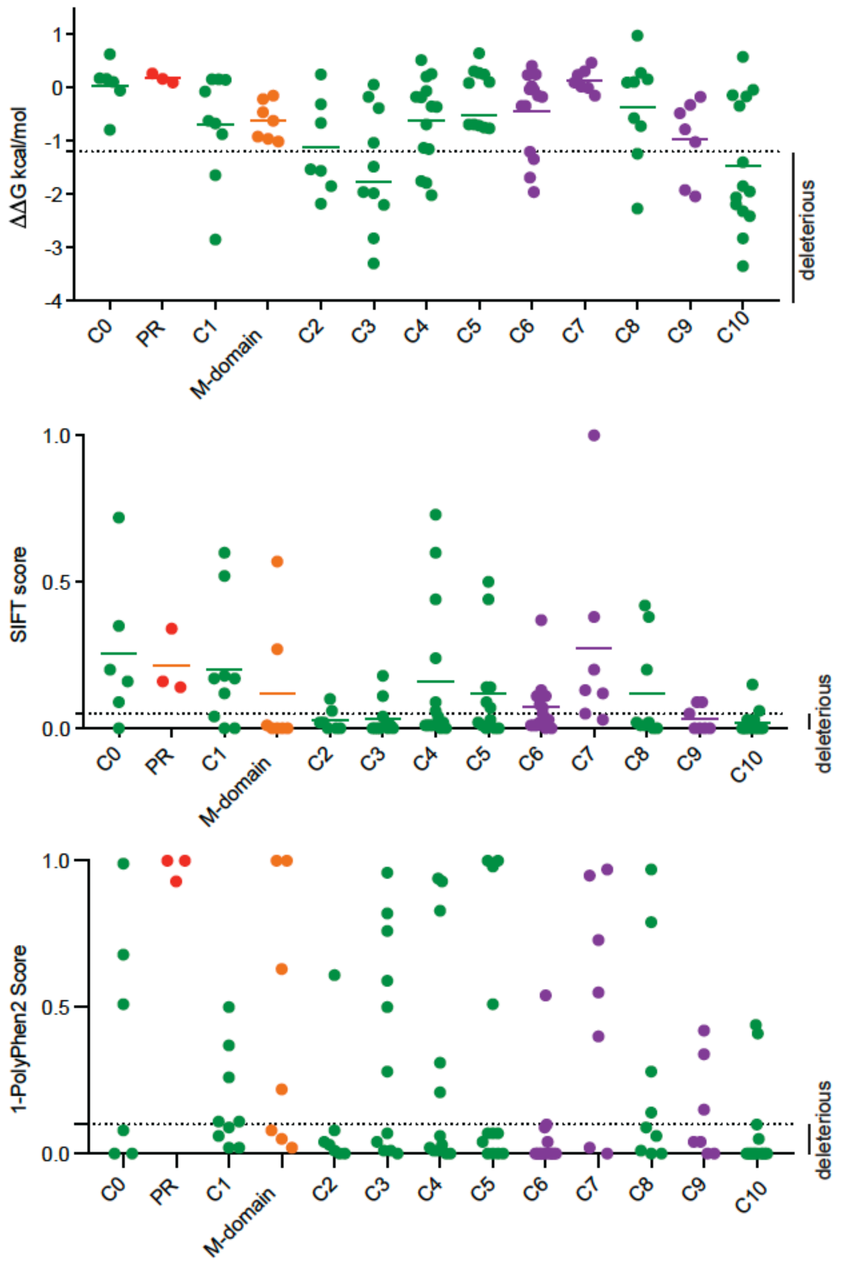

Figure S4: Computational analysis of missense *MYBPC3* VUSs. Missense *MYBPC3* VUS within SHaRe (Supplemental Table 2) were analyzed by STRUM (top panel), SIFT (middle panel) and PolyPhen-2 (lower panel). Results are grouped by individual subdomains with immunoglobulin domains (green; C0, C1, C2, C3, C4, C5, and C8) fibronectin domains (purple; C6, C76, C9). The linker regions - proline rich (PR, red) and M-domain (orange) linkers. The cut-off for likely deleterious variants (SIFT <

or = 0.05, 1- PolyPhen-2 < 0.10) is designated by dotted line with region below representing variants predicted to be deleterious.

In the STRUM analysis, the proportion of *MYBPC3* VUSs predicted to be deleterious varied by subdomain. For example, a higher proportion of VUSs were predicted to be deleterious within the C3 (63%) C2 (57%) and C10 domains (38%) compared to other immunoglobulin domains. High quality ITASSER models were obtained for each subdomain (Supplemental Figure 1). Of note very few variants within SHaRe were present within linker regions outside of defined subdomains (4%) (Supplemental Table 1,2). Two linker domains within MYBP-C accounted for the majority of linker region variants (22/24) and were modeled (Supplemental Figure 2-3). The PR domain was predicted to be a flexible and largely unstructured linker whereas the M-domain was predicted to exhibit beta-sheet secondary structure (Supplemental Table 3, Supplemental Figure 2). *MYBPC3* VUSs within the PR linker were consistently predicted to be non-deleterious not only by STRUM but also by the sequence-based algorithms Polyphen-2 and SIFT (Supplemental Figure 4).

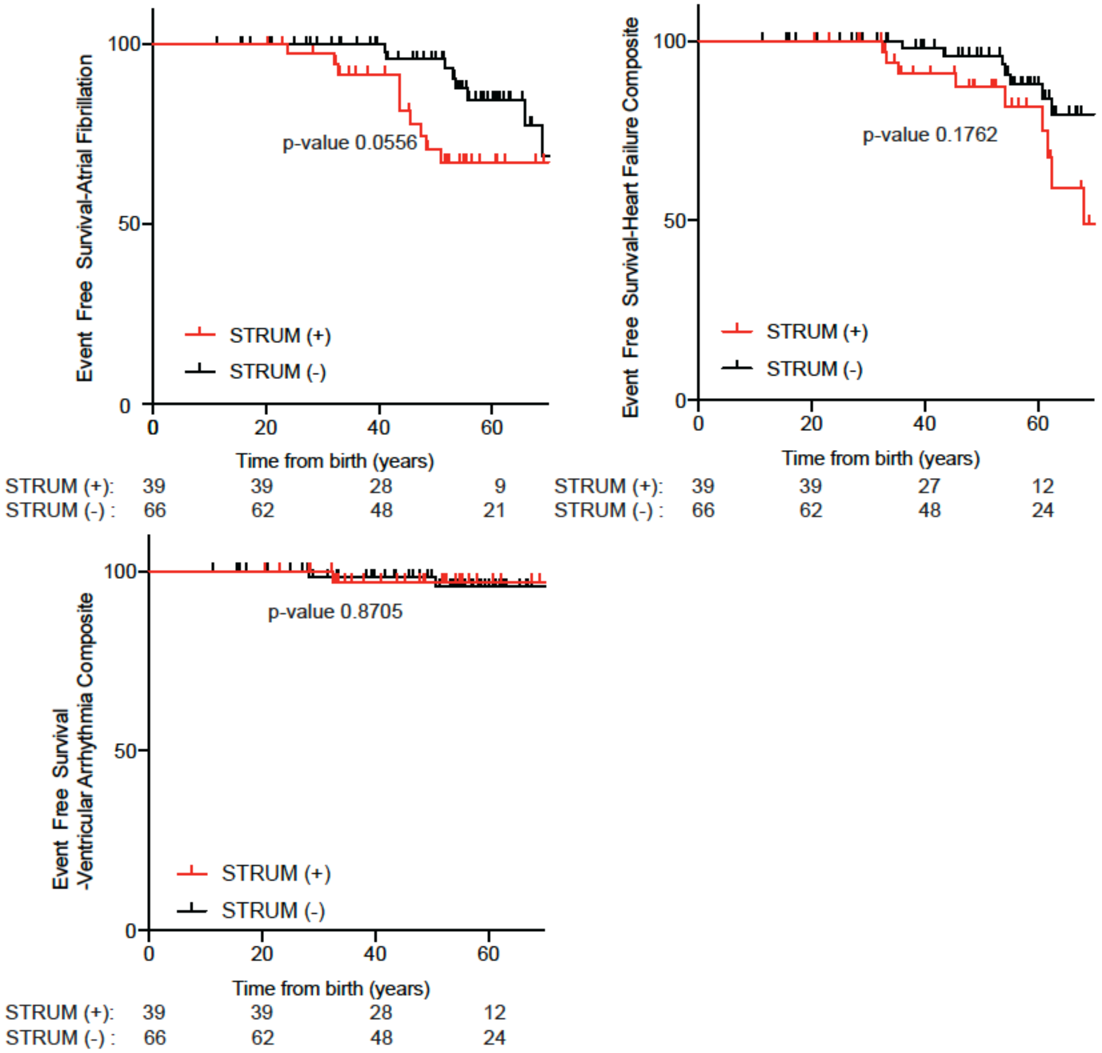

Figure S5: Risk stratification of individual composite outcomes. Variants predicted to be deleterious by STRUM (STRUM+, ΔΔG ≤ -1.2 kcal/mol, red, n =39) did exhibit higher rates of atrial fibrillation and heart failure composite than variants not predicted to be deleterious STRUM-, black, n=66). However, we could not exclude the null hypothesis (p -value 0.0559 and 0.1762). No difference in ventricular arrhythmia composite was observed. As previous described[8] composite outcomes reported are defined as follows;

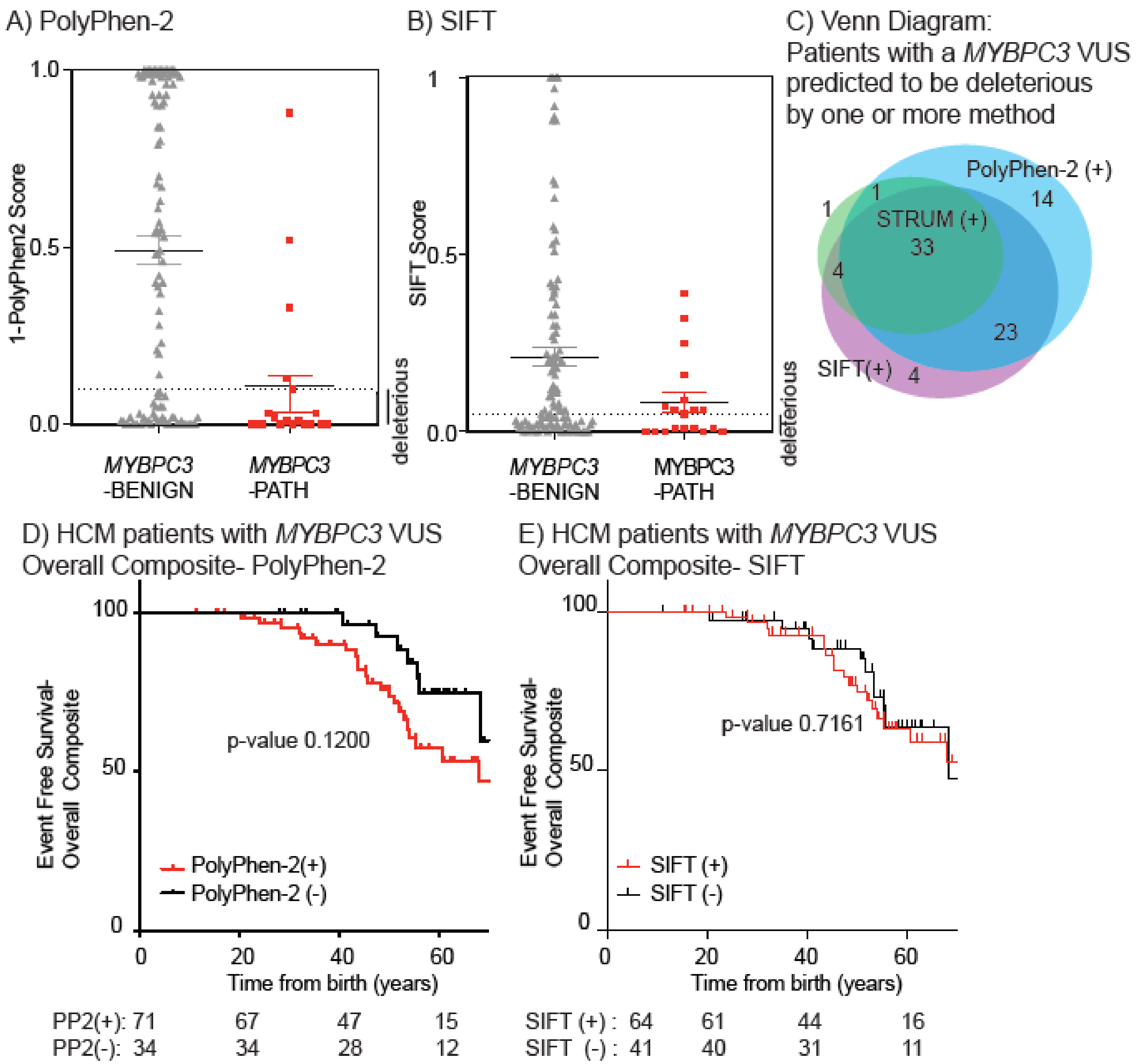

A) PolyPhen-2
1-PolyPhen2 Score
1.0
0.5
0.0
deleterious
MYBPC3 -BENIGN
MYBPC3 -PATH
B) SIFT
SIFT Score
1
0.5
0.0
deleterious
MYBPC3 -BENIGN
MYBPC3 -PATH
C) Venn Diagram: Patients with a MYBPC3 VUS predicted to be deleterious by one or more method
PolyPhen-2 (+)
14
1
1
STRUM (+)
33
4
23
SIFT(+)
4
D) HCM patients with MYBPC3 VUS Overall Composite- PolyPhen-2
Event Free Survival- Overall Composite
100
50
0
p-value 0.1200
PolyPhen-2(+)
PolyPhen-2 (-)
0
20
40
60
Time from birth (years)
PP2(+): 71 67 47 15
PP2(-): 34 34 28 12
E) HCM patients with MYBPC3 VUS Overall Composite- SIFT
Event Free Survival- Overall Composite
100
50
0
p-value 0.7161
SIFT (+)
SIFT (-)
0
20
40
60
Time from birth (years)
SIFT (+) : 64 61 44 16
SIFT (-) : 41 40 31 11

Figure S6: Sequence-based algorithms were exhibit decreased specificity compared to STRUM and are unable to identify a subgroup of HCM patients with a *MYBPC3* VUS at increased risk for HCM-related adverse clinical outcomes.

Results of computational analysis for each unique *MYBPC3*-Benign (grey triangles, n= 110) and *MYBPC3*-Path (red circles, n =19) variant are shown. Mean and SEM for each group depicted. The cut-off for deleterious variants were (A) Polyphen-2 > 0.90 (graphed as 1-Polyphen-2 score < 0.10) and (B) SIFT ≤ 0.10. Both SIFT and Polyphen-2 demonstrated decreased specificity 62% and 54% respectively compared to STRUM 93% (Figure 3). (C) We next evaluated patients with HCM and a MYBPC3 VUS. A ven diagram is shown for patients with HCM and a MYBPC3 VUS predicted to be deleterious by one or more of the following algorithms; SIFT, PolyPhen-2, STRUM. (D) Variants predicted to be deleterious by PolyPhen-2 (PolyPhen-2 > 0.90, red, n =71)[28] did exhibit higher rates of adverse HCM-related clinical outcomes (Overall composite) compared to variants not predicted to be deleterious (black, n = 34) by Kaplan Meier event free survival analysis. However, we could not exclude the null hypothesis (p-value 0.1200). As defined previously, the overall composite[8] is defined as the first occurrence of any component of the ventricular arrhythmic or heart failure composite end point (without inclusion of LV ejection fraction), all- cause mortality, atrial fibrillation (AF), stroke, or death. (E) Variants predicted to be deleterious by SIFT[29] (SIFT score ≤0.05, red, n =64) and non-deleterious (black, n =41) exhibited similar rates of adverse HCM-related clinical outcomes.

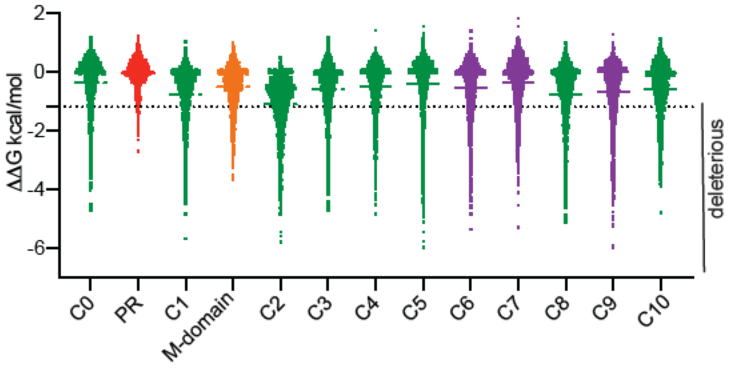

Figure S7: STRUM Analysis of *MYBPC3* VUSs. All possible MyBP-C missense and synonymous variants were analyzed by STRUM by performing *in silco* saturation mutagenesis (Supplemental Table 5). Subdomains of MyBP-C (the protein encoded by *MYBPC3*) are labeled and colored by domain type - immunoglobulin (C0, C1, C2, C3, C4, C5, and C8) in green, fibronectin (C6, C6, C9) in purple, and the proline rich (PR) and M-domain linkers in red and orange respectively. Horizontal line indicates mean for each group. Variants with ΔΔG ≤-1.2 kcal/mol are predicted to be deleterious. The cluster of variants at zero represent largely synonymous variants.

Table S1: Computational analysis of MYBPC3 non-synonymous missense variants – Benign and Path

See attached TableS1_ADT.xls

Table S2: Computational analysis of MYBPC3 non-synonymous missense variants- VUS

See attached TableS2_ADT.xlsx

Table S3: Summary of STRUM inputs and resulting I-TASSER models

MyBP-C Domain	Model Input Sequence	Domain Type	STRUM A.A.	Available Structures	C-score	Estimated TM score	RMSD
C0	2 to 96	Ig-like C2	2-96	2K1M (NMR)[18]	0.32	0.76+/-0.10	0.29
C1	153 to 256	Ig-like C2	153-256	2AVG (NMR)[17]	0.22	0.74+/- 0.11	0.44
				3CX2 (Xray)[20]			1.35
				2V6H (Xray)[19]			1.35
C2	362 to 452	Ig-like C2	362-452	1PD6 (NMR)[16]	0.42	0.77+/-0.10	0.30
				5K6P (NMR)[21]			1.99
C3	453-543	Ig-like C2	453-543	2MQ0 (NMR)[22]	0.59	0.79+/- 0.09	0.40
C4	542-633	Ig-like C2	544-633	none	0.4	0.77+/-0.10	NA
C5	645-771	Ig-like C2	645-771	1GXE (NMR)[23]	0.34	0.76+/- 0.10	0.48
C6	774-870	Fibronectin	774-870	none	0.90	0.84+/- 0.08	NA
C7	872-967	Fibronectin	872-967	none	0.98	0.85+/-0.08	NA
C8	970-1065	Ig-like C2	970-1065	none	0.44	0.77+/-0.10	NA
C9	1068-1163	Fibronectin	1068-1163	none	0.60	0.79+/- 0.09	NA
C10	1181-1274	Ig-like C2	1181-1274	none	0.77	0.82+/-0.09	NA
C0-PR-C1	1 to 260	NA	97-152	2K1M (NMR)	-2.01	0.47+/-0.15	1.39
				2AVG (NMR)			1.61
				3CX2 (Xray)			1.41
				2V6H (Xray)			1.20
C1-M-C2	152 to 452	NA	257-361	2AVG (NMR)	-0.72	0.62+/-0.14	2.02
				3CX2 (Xray)			1.07
				2V6H (Xray)			1.19
				1PD6 (NMR)			2.78
				5K6P (NMR)			3.38

The C-score range is from [-5,2] where a C-score of higher value signifies a model with high confidence. Another measure of model quality is TM-score, that is sensitive to local error. A TM-score > 0.5 indicates a model of correct topology. Our models exhibit TM-score of 0.74- 0.84. Finally, we report the root mean square deviation (RMSD) in Angstroms comparing I-TASSER models to known *MYBPC3* subdomain structures. STRUM A.A. indicates the amino acids (A.A) whom utilized the a given model input sequence to execute the STRUM analysis.

Table S4. STRUM results by subdomain

MyBP-C		ΔΔG kcal/mol		Variants	% deleterious
Domain	Domain Type	Mean	Stdev	Analyzed	ΔΔG ≤-1.2 kcal/mol
C0	IgG-like C2	-0.37	0.90	1805	15.79
PR	linker	-0.04	0.47	1120	2.50
C1	IgG-like C2	-0.78	0.96	2080	25.34
M domain	linker	-0.51	0.74	2100	17.05
C2	IgG-like C2	-1.11	0.99	1820	35.49
C3	IgG-like C2	-0.59	0.89	1820	19.51
C4	IgG-like C2	-0.50	0.87	1800	16.78
C5	IgG-like C2	-0.43	1.01	2540	16.50
C6	Fibronectin	-0.53	0.95	1940	19.48
C7	Fibronectin	-0.34	0.89	1920	15.83
C8	IgG-like C2	-0.79	1.06	1920	26.61
C9	Fibronectin	-0.69	1.09	1920	22.40
C10	IgG- like C2	-0.58	0.93	1880	21.28

Table S5: Complete STRUM analysis of MYBPC3 Missense Variants

See attached TableS5_ADT.xls

References:

1. Helms AS, Thompson AD, Glazier AA, Hafeez N, Kabani S, Rodriguez J, et al. Spatial and Functional Distribution of MYBPC3 Pathogenic Variants and Clinical Outcomes in Patients With Hypertrophic Cardiomyopathy. Circ Genom Precis Med. 2020;13(5):396-405.
2. Richards S, Aziz N, Bale S, Bick D, Das S, Gastier-Foster J, et al. Standards and guidelines for the interpretation of sequence variants: a joint consensus recommendation of the American College of Medical Genetics and Genomics and the Association for Molecular Pathology. Genet Med. 2015;17(5):405-24. doi: 10.1038/gim.2015.30. Epub Mar 5.
3. Ito K, Patel PN, Gorham JM, McDonough B, DePalma SR, Adler EE, et al. Identification of pathogenic gene mutations in LMNA and MYBPC3 that alter RNA splicing. Proc Natl Acad Sci U S A. 2017;114(29):7689-94.
4. Carrier L, Mearini G, Stathopoulou K, Cuello F. Cardiac myosin-binding protein C (MYBPC3) in cardiac pathophysiology. Gene. 2015;573(2):188-97.
5. Singer ES, Ingles J, Semsarian C, Bagnall RD. Key Value of RNA Analysis of MYBPC3 Splice-Site Variants in Hypertrophic Cardiomyopathy. Circ Genom Precis Med. 2019;12(1):e002368.
6. Richards S, Aziz N, Bale S, Bick D, Das S, Gastier-Foster J, et al. Standards and guidelines for the interpretation of sequence variants: a joint consensus recommendation of the American College of Medical Genetics and Genomics and the Association for Molecular Pathology. Genet Med. 2015;17(5):405-24.
7. Kelly MA, Caleshu C, Morales A, Buchan J, Wolf Z, Harrison SM, et al. Adaptation and validation of the ACMG/AMP variant classification framework for MYH7-associated inherited cardiomyopathies: recommendations by ClinGen's Inherited Cardiomyopathy Expert Panel. Genet Med. 2018;20(3):351-9.

8. Ho CY, Day SM, Ashley EA, Michels M, Pereira AC, Jacoby D, et al. Genotype and Lifetime Burden of Disease in Hypertrophic Cardiomyopathy: Insights from the Sarcomeric Human Cardiomyopathy Registry (SHaRe). Circulation. 2018;138(14):1387-98.

9. Toepfer CN, Garfinkel AC, Venturini G, Wakimoto H, Repetti G, Alamo L, et al. Myosin Sequestration Regulates Sarcomere Function, Cardiomyocyte Energetics, and Metabolism, Informing the Pathogenesis of Hypertrophic Cardiomyopathy. Circulation. 2020;141(10):828-42.

10. Zhang X.; Walsh R.; Whiffin N.; Buchan R.; Midwinter W. WAGR, Li N.; Ahmad M.; Mazzarotto F.; Roberts, A.; Theotokis, P.I.; Mazaika, E.; Allouba, M.; de Marvao, A.; Pua, C.J.; Day, S.M.; Ashley E., Colan, S.D., Michels, M.; Pereira A.C.; Jacoby, D.; Ho, C.Y.; Olivotto, I.; Gunnarsson, G.T.; Jefferies, J.L.; Semsarian, C.; Ingles, J.; O'Regan, D. P.; Aguib, Y.; Yacoub, M.H.; Cook, S.A.; Barton, P.J.R.; Bottolo, L.; Ware, J.S. Disease-specific variant pathogenicity prediction significantly improves variant interpretation in inherited cardiac conditions. . Genetics in Medicine 2020;Published online: 13 Oct 2020

11. Quan L, Lv Q, Zhang Y. STRUM: structure-based prediction of protein stability changes upon single-point mutation. Bioinformatics. 2016;32(19):2936-46.

12. Yang J, Yan R, Roy A, Xu D, Poisson J, Zhang Y. The I-TASSER Suite: protein structure and function prediction. Nat Methods. 2015;12(1):7-8.

13. Yang J, Zhang Y. I-TASSER server: new development for protein structure and function predictions. Nucleic Acids Res. 2015;43(W1):W174-81.

14. Roy A, Kucukural A, Zhang Y. I-TASSER: a unified platform for automated protein structure and function prediction. Nat Protoc. 2010;5(4):725-38.

15. Lee K, Harris SP, Sadayappan S, Craig R. Orientation of myosin binding protein C in the cardiac muscle sarcomere determined by domain-specific immuno-EM. J Mol Biol. 2015;427(2):274-86.

16. Ababou A, Gautel M, Pfuhl M. Dissecting the N-terminal myosin binding site of human cardiac myosin-binding protein C. Structure and myosin binding of domain C2. J Biol Chem. 2007;282(12):9204-15.

17. Ababou A, Rostkova E, Mistry S, Le Masurier C, Gautel M, Pfuhl M. Myosin binding protein C positioned to play a key role in regulation of muscle contraction: structure and interactions of domain C1. J Mol Biol. 2008;384(3):615-30.

18. Ratti J, Rostkova E, Gautel M, Pfuhl M. Structure and interactions of myosin-binding protein C domain C0: cardiac-specific regulation of myosin at its neck? J Biol Chem. 2011;286(14):12650-8.

19. Govada L, Carpenter L, da Fonseca PC, Helliwell JR, Rizkallah P, Flashman E, et al. Crystal structure of the C1 domain of cardiac myosin binding protein-C: implications for hypertrophic cardiomyopathy. J Mol Biol. 2008;378(2):387-97.

20. Fisher SJ, Helliwell JR, Khurshid S, Govada L, Redwood C, Squire JM, et al. An investigation into the protonation states of the C1 domain of cardiac myosin-binding protein C. Acta Crystallogr D Biol Crystallogr. 2008;64(Pt 6):658-64.

21. Michie KA, Kwan AH, Tung CS, Guss JM, Trewhella J. A Highly Conserved Yet Flexible Linker Is Part of a Polymorphic Protein-Binding Domain in Myosin-Binding Protein C. Structure. 2016;24(11):2000-7.

22. Zhang XL, De S, McIntosh LP, Paetzel M. Structural characterization of the C3 domain of cardiac myosin binding protein C and its hypertrophic cardiomyopathy-related R502W mutant. Biochemistry. 2014;53(32):5332-42.

23. Idowu SM, Gautel M, Perkins SJ, Pfuhl M. Structure, stability and dynamics of the central domain of cardiac myosin binding protein C (MyBP-C): implications for multidomain assembly and causes for cardiomyopathy. J Mol Biol. 2003;329(4):745-61.

24. Squire JM, Luther PK, Knupp C. Structural evidence for the interaction of C-protein (MyBP-C) with actin and sequence identification of a possible actin-binding domain. J Mol Biol. 2003;331(3):713-24.

25. Oakley CE, Hambly BD, Curmi PM, Brown LJ. Myosin binding protein C: structural abnormalities in familial hypertrophic cardiomyopathy. Cell Res. 2004;14(2):95-110.

26. Moolman-Smook J, Flashman E, de Lange W, Li Z, Corfield V, Redwood C, et al. Identification of novel interactions between domains of Myosin binding protein-C that are modulated by hypertrophic cardiomyopathy missense mutations. Circ Res. 2002;91(8):704-11.

27. Flashman E, Redwood C, Moolman-Smook J, Watkins H. Cardiac myosin binding protein C: its role in physiology and disease. Circ Res. 2004;94(10):1279-89.

28. Adzhubei I, Jordan DM, Sunyaev SR. Predicting functional effect of human missense mutations using PolyPhen-2. Curr Protoc Hum Genet. 2013;Chapter 7:Unit7 20.

29. Sim NL, Kumar P, Hu J, Henikoff S, Schneider G, Ng PC. SIFT web server: predicting effects of amino acid substitutions on proteins. Nucleic Acids Res. 2012;40(Web Server issue):W452-7.

30. Walsh R, Mazzarotto F, Whiffin N, Buchan R, Midwinter W, Wilk A, et al. Quantitative approaches to variant classification increase the yield and precision of genetic testing in Mendelian diseases: the case of hypertrophic cardiomyopathy. Genome Med. 2019;11(1):5.
